# Updated normative data for the EORTC QLQ-C30 in the general Dutch population by age and sex: a cross-sectional panel research study

**DOI:** 10.1007/s11136-023-03404-2

**Published:** 2023-04-09

**Authors:** K. M. de Ligt, N. K. Aaronson, G. Liegl, S. Nolte, the EORTC Quality of Life Group

**Affiliations:** 1 Division of Psychosocial Research and Epidemiology, Netherlands Cancer Institute, Amsterdam, The Netherlands; 2 Patient-Centred Outcomes Research, Medical Clinic, Department of Psychosomatic Medicine, Charité – Universitätsmedizin Berlin, Corporate Member of Freie Universität Berlin and Humboldt-Universität Zu Berlin, Berlin, Germany

**Keywords:** Oncology, Health-related quality of life, Patient-reported outcomes, Patient-reported outcome measures, Normative data, EORTC QLQ-C30, Netherlands

## Abstract

**Purpose:**

The European Organisation for Research and Treatment of Cancer (EORTC) quality of life core questionnaire (QLQ-C30) is a validated and widely-used Patient-Reported Outcome Measure for measuring the health-related quality of life (HRQoL) of cancer patients. To facilitate interpretation of results obtained in studies using the EORTC QLQ-C30, we generated normative data for the Dutch general population, stratified by age and sex.

**Methods:**

Dutch participants were selected from a larger cross-sectional online panel research study collecting EORTC QLQ-C30 general population normative data across 15 countries. EORTC QLQ-C30 raw scores based on a 4-point response scale were transformed to linear scores ranging from 0 to 100. Transformed scores were weighted based on the United Nations population distribution statistics and presented by age and sex/age. Differences in scale scores of ≥ 10 points in HRQoL were applied to indicate clinical relevance.

**Results:**

One thousand respondents completed the online survey. Stratified by age, clinically meaningful differences were observed, with worse physical functioning scores and better emotional functioning scores with increased age. Symptom scores remained stable across age groups, except for small age differences observed for fatigue, nausea/vomiting, diarrhoea, and financial difficulties. Stratified by sex/age, men generally scored better for both functioning and symptoms. However, these differences were not clinically meaningful.

**Conclusions:**

These updated normative EORTC QLQ-C30 for the Dutch general population can be used to better interpret HRQoL data obtained from Dutch cancer patients. Being part of a larger international study, these data can further be used for inter-country comparisons in multi-national studies.

## Plain language summary

Patient-Reported Outcome Measures (PROMs) are questionnaires completed by patients to measure their quality of life. The European Organisation for Research and Treatment of Cancer (EORTC) Quality of Life Core Questionnaire, the QLQ-C30, is a questionnaire widely used for cancer patients. To help interpret the scores that result from this questionnaire, scores can be compared with the scores from the general population, so-called normative scores. This study generated updated EORTC QLQ-C30 data for the Dutch general population that had been collected as part of a large European Norm Data project. This is important, because national normative data allows for more meaningful interpretation of scores within a specific country. Scores are presented separately for men and women, and for different age groups. We found that with increasing age, worse physical but better emotional functioning was reported. Men generally reported better functioning and fewer symptoms compared to women. The normative EORTC QLQ-C30 data for the Dutch general population can be used to better interpret data obtained from Dutch cancer patients.

## Background

Patient-Reported Outcome Measures (PROMs), designed to quantify patients’ experiences with disease and treatment, have been increasingly applied in oncology trials and observational studies over the past decades [1]. The European Organisation for Research and Treatment of Cancer (EORTC) Quality of Life Core Questionnaire, the QLQ-C30, is a validated PROM available in many languages. The EORTC QLQ-C30 assesses 15 domains of functional health and symptoms commonly experienced by cancer patients [2]. It is widely used in clinical trials and as part of clinical research [3]. Nowadays, PROMs are also increasingly integrated into daily clinical practice for assessing and managing health-related quality of life (HRQoL) in individual patients [4, 5], following the paradigm shift to patient-centred healthcare [5, 6]. The International Consortium for Health Outcomes Measurement (ICHOM) recommends the adoption of the EORTC QLQ-C30 in clinical practice, for example, for patients with breast [7], lung [8], advanced prostate [9], and colorectal cancer [10].

An important strategy to aid in the interpretation of cancer patients’ HRQoL is to compare these with general population scores, so-called normative data [11]. In 2017, normative data for the original EORTC QLQ-C30 and its computer-adaptive test version (EORTC CAT Core [12]) were systematically collected via an online research panel in the general populations of 11 European Union (EU) countries, and Russia, Turkey, Canada, and the United States in a large international study [13, 14]. To date, this has resulted in two core publications [13], 14 and separate national norm data papers for the German [15], Austrian [16], Spanish [17] and Italian [18] general populations, respectively. For studies conducted at the local or national level, it is important to have country-specific normative data available, because normative data can vary between countries and cultures [13]. Thus, national normative data allows for more meaningful score interpretation within a specific country.

Van de Poll-Franse et al. [19] published Dutch EORTC QLQ-C30 data for the general population based on a 2009 survey of 1700 individuals. Subsequently, Mols et al. [20] carried out five consecutive annual assessments (2009–2013) of the EORTC QLQ-C30 in 1700–2400 individuals from the Dutch general population. However, these studies were not conducted in line with other international general population studies. To enable inter-country comparison, the aim of the present study was to generate and describe updated EORTC QLQ-C30 data for the Dutch general population, based on the 2017 EORTC international online survey that employed a uniform sampling strategy across participating countries [13, 14]. Results were stratified by age and sex, both having a distinct impact on HRQoL [21, 22]. These updated normative data will facilitate the interpretation of HRQoL data within the Netherlands, and their comparison with data obtained in other countries.

## Materials and methods

### Sampling and setting

Dutch general population data were retrieved from an international online cross-sectional study undertaken in March/April 2017, in which participants completed a survey about HRQoL; for further details on the data collection methods, please see Nolte et al. [13] and Liegl et al. [14]. In ten strata based on sex and age group (male/female; 18–39, 40–49, 50–59, 60–69, ≥70 years), an equal number of participants was recruited to meet the recruitment quota (anticipated sample size: *n* = 100/stratum). Participants were voluntary online panel members who gave consent to participate in panel-based studies. Data were collected by the panel research company GfK SE (https://www.gfk.com/). For a given country, panels are representative for the general population with internet access based on criteria including age, sex, education, household size, city size, and geographical location. Response rates to panel studies conducted by GfK are generally between 75 and 90% [13].

### Measures

Participants were asked to complete a questionnaire that, among other data (for details, see Liegl et al. [14]) contained the following:

1. The EORTC QLQ-C30 [2]: 30 items measuring 15 scales: functioning (physical, role, emotional, cognitive, and social), symptoms (fatigue, nausea/vomiting, pain, dyspnoea, insomnia, appetite loss, constipation, diarrhoea, and financial difficulties) and global HRQoL. Response options were on a 4-point Likert-type scale (not at all; a little; quite a bit; very much) and a 7-point Likert scale for global HRQoL ranging from ‘Very poor’ to ‘Excellent’. The recall period was the previous week, with the exception of the physical functioning scale that refers to the current situation. Participants completed the Dutch version of the EORTC QLQ-C30, which has been shown to be reliable and valid for use in the Netherlands [2]. Following standard scoring procedures, raw scores are transformed linearly to scale scores ranging from 0 to 100, with higher scores indicating better global HRQoL and functioning, but worse symptom experience [23];
2. Sociodemographic items, including sex, age, educational level, employment status, relationship status, and prevalence of health conditions.

### Statistical analyses

The purpose of collecting norm data from the general population is to guide interpretation of HRQoL data obtained from cancer patients [11]. Therefore, we provide look-up tables where the norm data are presented descriptively, further stratified by age and sex. Differences of ≥ 10 points were considered clinically relevant [28]. In those cases where clinically meaningful group differences by age and/or sex were found, we further calculated effect sizes (Cohen’s *d*) to better understand the size of detected differences. First, sociodemographic data and EORTC QLQ-C30 mean scale scores and standard deviations (SD) were described. The EORTC QLQ-C30 summary score, available since 2016, was the mean of all EORTC QLQ-C30 scale scores (excluding global QoL and financial difficulties); the Summary score ranges from 0 to 100, with higher scores indicating better HRQoL [24]. It has a strong prognostic value for overall survival for cancer populations, and additional value beyond traditional clinical and sociodemographic variables [25].

Second, to achieve norm scores to be representative for the Dutch general population, mean scale scores were weighted by the sex and age distribution of the adult Dutch population [26], using the 2015 population distribution statistics published by the United Nations, Department of Economic and Social Affairs, Population Division [27]. The sample was not weighted for health conditions because such information was not available. Of note, akin to the other norm data papers [15–18], we further divided the youngest age group (18–39 years) into two groups, i.e., 18–29 years and 30–39 years. All analyses were performed in Stata/SE 14.2 [29].

## Results

### Sample population

Unweighted and weighted sample population characteristics are presented in Table 1, with unweighted data presented here. The sample consisted of 499 men and 501 women (*N* = 1000). Their mean age was 54.0 years (SD: 15.3 years), 42% completed university or a post-graduate degree, and 73% were married or in a stable relationship. Thirty percent were employed full-time, 19% part-time and 31% were retired. Half of them reported having at least one health condition. The most frequently reported health conditions were chronic pain (15%), arthritis (10%), diabetes (8%), and heart disease (6%); 2.3% reported a diagnosis of cancer.

**Table 1:** Sample characteristics (*N* = 1000), unweighted and weighted data

	Unweighted data^a^						Weighted data^a,b^					
	Full sample (*N* = 1000)		Males (*n* = 499)		Female (*n* = 501)		Full sample (*n* = 1000)		Males (*n* = 493.8)		Female (*n* = 506.2)	
	*n*	%	*n*	%	*n*	%	*n*	%	*n*	%	*n*	%
**Age (years)**												
*Mean (SD)*	54.0	(15.3)	54.4	(15.1)	(53.6)	(15.6)	47.9	(17.2)	47.4	(17.0)	48.3	(17.4)
*Categories (in years)*												
18–29	74	7.4	30	6.0	44	8.8	222.6	22.3	113.4	23.0	109.2	21.6
30–39	124	12.4	68	13.6	56	11.2	143.0	14.3	72.0	14.6	71.0	14.0
40–49	200	20.0	100	20.0	100	20.0	172.7	17.3	86.6	17.5	86.1	17.0
50–59	201	20.1	100	20.0	101	20.2	172.1	17.2	86.9	17.6	85.2	16.8
60–69	200	20.0	100	20.0	100	20.0	146.9	14.7	73.1	14.8	73.9	14.6
70+	201	20.1	101	20.2	100	20.0	142.7	14.3	61.8	12.5	80.9	16.0
**Education**												
Less than compulsory education	0	0	0	0	0	0	0	0	0	0	0	0
Compulsory (left school at the minimum school leaving age)	12	1.2	7	1.4	5	1.0	11.5	1.2	5.7	1.2	5.8	5.8
Some post compulsory (some school after reaching school leaving age without reaching university entrance qualifications	108	10.9	39	7.9	69	13.9	103.8	10.5	39.9	8.1	63.9	12.7
Post compulsory below university (e.g. reaching A levels)	451	45.5	221	44.7	230	46.4	431.1	43.5	214.1	43.7	217.0	43.2
University degree (Bachelor’s or equivalent level)	303	30.6	163	33.0	140	28.2	302.3	30.5	157.1	32.1	145.2	28.9
Postgraduate degree (Master’s, Doctorate or equivalent level)	116	11.7	64	13.0	52	10.5	143.0	14.4	73.1	14.9	70.0	14.0
Prefer not to answer^c^	10	–	5	–	5	–	8.3	–	3.9	–	4.4	–
**Marital status**												
Single/not in a steady relationship	180	18.1	81	16.4	99	19.9	229.2	23.1	111.2	22.8	118.0	23.5
Married or in a steady relationship	727	73.2	389	78.6	338	67.9	693.2	70.0	357.4	73.3	335.8	66.7
Separated/divorced/widowed	86	8.7	25	5.1	61	12.2	68.4	6.9	19.0	19.0	49.5	9.8
Prefer not to answer^c^	7	–	4	–	3	–	9.2	–	6.3	–	2.9	–
**Employment status**												
Employed full-time	294	29.6	217	43.7	77	15.5	339.9	34.2	245.5	49.9	94.4	18.8
Employed part-time	186	18.7	41	8.2	145	29.2	198.5	20.0	50.3	10.2	148.2	29.5
Homemaker	32	3.2	1	0.2	31		28.9	2.9	1.1	0.2	27.9	5.6
Student	23	2.3	5	1.0	18	3.6	60.7	6.1	18.9	3.8	41.8	8.3
Unemployed	60	6.0	28	5.6	32	6.5	60.9	6.1	29.2	5.9	31.7	6.3
Retired	308	31.0	158	31.8	150	30.2	222.2	22.4	104.6	21.3	117.6	23.4
Self-employed	41	4.1	27	5.4	14	2.8	37.7	3.8	25.4	5.2	12.3	2.5
Other (please specify)	49	4.9	20	4.0	29	5.8	45.3	4.6	17.1	3.5	28.2	5.6
Prefer not to answer^c^	7	–	2	–	5	–	5.7	–	1.7	–	4.0	–
**Health status**												
I have no health condition/disease	479	47.9	265	53.1	214	42.7	518.7	51.9	286.8	58.1	231.8	45.8
At least one health condition^d^	521	52.1	234	46.9	287	57.3	481.3	48.1	207.0	41.9	274.4	54.2
Chronic pain (for example, low back pain, neck pain)	139	14.5	53	11.0	86	18.0	129.0	13.4	48.1	10.1	80.8	16.7
Heart disease (for example, coronary heart disease, heart attack, heart failure)	54	5.6	40	8.3	14	2.9	41.1	4.17	28.3	5.9	12.7	2.6
Cancer (excluding basal cell carcinoma)	22	2.3	10	2.0	12	2.5	16.4	1.7	6.9	1.5	9.5	2.0
Depression	36	3.7	18	3.7	18	3.8	42.7	4.4	23.8	5.0	18.9	3.9
Chronic Obstructive Pulmonary Disease (COPD)	41	4.3	24	4.9	17	3.5	30.2	3.1	16.7	3.5	13.5	2.8
Arthritis (for example, osteoarthritis, rheumatoid arthritis)	97	10.1	31	6.4	66	13.8	75.9	7.9	23.7	5.0	52.2	10.8
Diabetes	80	8.3	41	8.5	39	8.1	62.4	6.5	29.6	6.2	32.8	6.8
Asthma	52	5.4	21	4.3	31	6.5	57.4	6.0	21.0	4.4	36.4	7.5
Anxiety disorder	19	2.0	9	1.9	10	2.1	24.5	2.6	13.7	2.9	10.7	2.2
Obesity	28	2.9	8	1.7	20	4.2	27.4	2.9	7.0	1.5	20.4	4.2
Drug/alcohol use disorder	3	0.3	0	0	3	0.6	2.9	0.3	0	0.0	2.9	0.6
Other	148	15.4	61	12.7	87	18.2	139.1	14.5	50.4	10.6	88.7	18.3
Prefer not to answer^c^	40	–	18	–	22	–	40	–	18	–	22	–

*M* mean, *SD* standard deviation, *IQR* interquartile range

^a^Unless stated otherwise, the numbers refer to sample size (n) and percentage (%)

^b^Mean scale scores were weighted by the sex and age distribution of the adult Dutch population, using the 2015 population distribution statistics published by the United Nations Department of Economic and Social Affairs Population Division

^c^For calculating percentages, the category “prefer not to answer” was excluded from the denominator

^d^The sum of all health conditions is larger than the sample size as respondents could choose multiple response options

Weighting the population altered the age distribution slightly with more respondents in younger age categories, because the 18–39 years age group had a larger range compared to the 10-year range for the other age groups.

### EORTC QLQ-C30 scores

Weighted and unweighted EORTC QLQ-C30 scores are presented in Table 2. The weighing of data led to changes in scores ranging from -1.9 (emotional functioning) to +1.4 (fatigue).

**Table 2:** EORTC QLQ-C30 scores for the Dutch general population, weighted and unweighted scores

	Unweighted data		Weighted data^a^	
	Mean	SD	Mean	SD
Global health/HRQoL	78.4	19.1	77.4	19.8
**Functioning scales**				
Physical	89.7	15.5	90.7	14.9
Role	88.5	21.9	89.1	21.5
Emotional	84.2	19.2	82.3	21.2
Cognitive	90.4	16.1	90.3	17.1
Social	92.3	18.0	91.9	19.0
**Symptom scales**				
Fatigue	22.3	22.6	23.7	23.0
Nausea/vomiting	2.9	10.4	3.5	11.8
Pain	17.9	23.1	17.7	22.9
Dyspnoea	10.0	20.4	9.5	19.7
Insomnia	21.6	26.2	21.3	26.1
Appetite loss	4.9	15.2	4.9	15.1
Constipation	5.0	13.6	4.9	13.6
Diarrhoea	6.2	16.9	6.9	17.8
Financial difficulties	4.6	15.8	4.9	17.1
**Summary score**	88.8	11.9	88.6	12.1

^a^Mean scale scores were weighted by the sex and age distribution of the adult Dutch population, using the 2015 population distribution statistics published by the United Nations Department of Economic and Social Affairs Population Division

Weighted mean and SD for global health were 77.4 ± 19.8. Functioning scores ranged between 82.3 ± 21.2 for emotional functioning and 91.9 ± 19.0 for social functioning. Symptom scores ranged between 3.5 ± 11.8 for nausea/vomiting and 23.7 ± 23.0 for fatigue. For several symptom subscales (nausea/vomiting, dyspnoea, appetite loss, constipation, diarrhoea), floor effects were observed, as > 80% selected the best score. The weighted mean EORTC QLQ-C30 summary score was 88.6 ± 12.1.

### EORTC QLQ-C30 scores by age

Weighted EORTC QLQ-C30 scores stratified by age are presented in Table 3. Physical functioning was worse with increasing age (age 18–29 years: 93.9; age 70+ years: 84.9, Cohen’s *d*: = 0.65), although not clinically meaningful, while emotional functioning was better (clinically meaningful) with increasing age (age 18–29 years: 74.9; age 70+ years: 90.1, Cohen’s *d* = 0.67). Differences in other functioning scale scores were not clinically meaningful and relatively stable across age groups (Table 3). Most symptom scores remained stable across age groups. However, fatigue scores were worst in the youngest age group (age 18–29 years: 29.4), indicating a clinically meaningful difference between the youngest and the oldest age group (age 70+ years: 17.1, Cohen’s *d* = 0.56).

**Table 3:** Weighted EORTC QLQ-C30 scores^a^ for the Dutch general population stratified by age category (in years; men and women combined)

	Full sample		18–29 years		30–39 years		40–49 years		50–59 years		60–69 years		70+ years	
	Mean	SD	Mean	SD	Mean	SD	Mean	SD	Mean	SD	Mean	SD	Mean	SD
**Global health/quality of life**	77.4	19.8	73.4	22.0	75.5	20.7	76.6	20.9	79.4	16.6	78.6	19.5	83.1	16.1
**Functioning scales**														
Physical	90.7	14.9	93.9	11.4	93.7	12.5	91.6	15.9	91.0	14.3	87.0	16.7	84.9	16.8
Role	89.1	21.5	92.1	19.3	89.6	22.7	88.1	23.9	88.5	21.0	86.7	22.6	88.6	19.8
Emotional	82.3	21.2	74.9	27.1	79.3	21.4	82.4	21.2	83.4	17.1	87.3	17.0	90.1	13.5
Cognitive	90.3	17.1	89.0	21.3	90.3	18.3	90.1	17.5	90.9	15.7	91.3	13.4	90.5	12.9
Social	91.9	19.0	90.1	22.2	91.6	20.4	90.8	20.4	94.0	15.4	92.2	16.8	93.4	16.1
**Symptom scales**														
Fatigue	23.7	23.0	29.4	24.5	26.2	23.3	25.3	24.8	21.5	20.4	20.1	22.8	17.1	18.7
Nausea/vomiting	3.5	11.8	6.4	17.1	4.31	12.4	2.5	7.8	2.3	9.1	2.5	9.7	2.1	9.4
Pain	17.7	22.9	16.7	21.5	15.7	22.1	16.5	24.2	21.0	23.5	20.7	24.7	15.5	21.1
Dyspnoea	9.5	19.7	7.6	15.7	10.3	24.5	7.3	17.4	9.6	18.1	13.2	23.9	10.7	19.5
Insomnia	21.3	26.1	18.9	25.0	22.1	24.6	22.1	29.0	23.6	27.2	22.2	25.8	19.3	24.2
Appetite loss	4.9	15.1	5.3	14.9	5.4	15.4	4.8	15.8	3.6	13.2	4.8	14.4	5.7	16.8
Constipation	4.9	13.6	4.7	13.6	4.8	14.4	4.2	12.5	4.9	12.8	5.0	13.7	6.3	15.1
Diarrhoea	6.9	17.8	9.3	20.6	9.5	21.0	6.0	16.3	4.6	14.1	6.3	17.2	4.7	15.0
Financial difficulties	4.9	17.1	6.7	22.2	5.4	19.2	3.8	13.4	5.3	15.4	4.7	16.1	2.8	11.8
**Summary score**	88.6	12.1	87.8	12.4	88.2	13.7	88.8	13.5	89.0	10.7	88.4	11.8	89.7	10.0

^a^Mean scale scores were weighted by the sex and age distribution of the adult Dutch population, using the 2015 population distribution statistics published by the United Nations Department of Economic and Social Affairs Population Division

Mean Global HRQoL was better with increasing age (age 18–29 years: 73.4; age 70+ years: 83.1, although not clinically meaningful, Cohen’s *d* = 0.49), while the mean EORTC QLQ-C30 summary score was relatively stable across age categories (age 18–29 years: 87.8; age 70+ years: 89.7, Cohen’s *d* = 0.16).

### EORTC QLQ-C30 scores by sex and age

Scores stratified by age and sex are presented in Table 4 (men) and Table 5 (women). Generally, men had slightly more favourable scores compared to women: physical (men: 92.8, women: 88.6, Cohen’s *d* = 0.29), role (men: 90.7, women: 87.6, Cohen’s *d* = 0.14), social (men: 93.2, women: 90.7, Cohen’s *d* = 0.13), and emotional (men: 84.4, women: 80.2, Cohen’s *d* = 0.20) functioning, and global health (men: 79.3, women: 75.6, Cohen’s *d* = 0.19) were 3–4 points higher in men. Men reported more favourable symptom scores than women for insomnia (9.9-point difference, Cohen’s *d* = 0.39), pain (7.1-point difference, Cohen’s *d* = 0.31), fatigue (6.2-point difference, Cohen’s *d* = 0.27), and dyspnoea (4.4-point difference, Cohen’s *d* = 0.22). The mean EORTC QLQ-C30 summary score was again slightly more favourable for men (men: 90.7, women: 86.5, Cohen’s *d* = 0.35). None of these were clinically meaningful.

**Table 4:** Weighted EORTC QLQ-C30 scores^a^ for the Dutch general population stratified by age category (men)

	Full sample		18–29 years		30–39 years		40–49 years		50–59 years		60–69 years		70+ years	
	Mean	SD	Mean	SD	Mean	SD	Mean	SD	Mean	SD	Mean	SD	Mean	SD
**Global health/quality of life**	79.3	19.1	78.3	22.4	77.0	18.3	78.8	19.4	79.5	16.0	80.7	18.9	82.8	16.8
**Functioning scales**														
Physical	92.8	13.7	96.2	10.0	93.5	14.5	93.9	13.8	91.5	14.9	91.2	13.8	87.9	15.3
Role	90.7	20.3	95.0	13.8	91.2	23.5	89.7	22.1	87.7	22.1	89.7	20.2	89.1	20.8
Emotional	84.4	20.6	77.2	28.2	81.3	20.5	84.9	18.7	86.6	16.0	88.6	16.0	92.7	10.8
Cognitive	90.7	17.5	91.1	19.7	88.7	21.6	92.5	16.5	90.8	16.8	91.3	14.1	88.9	13.4
Social	93.2	18.7	91.1	24.3	92.6	20.6	94.2	15.2	94.0	16.9	93.2	16.3	94.9	13.9
**Symptom scales**														
Fatigue	20.6	20.6	23.3	17.8	25.2	24.5	21.2	22.0	20.2	20.6	16.0	20.0	15.4	17.8
Nausea/vomiting	2.3	8.8	3.9	12.7	2.7	9.4	1.3	5.1	2.2	8.8	1.3	5.1	1.5	6.3
Pain	14.1	20.1	11.7	16.8	14.5	21.3	13.8	22.4	18.2	22.5	15.8	19.8	10.9	17.1
Dyspnoea	7.4	17.8	3.3	10.0	9.8	23.8	5.3	14.0	7.7	16.3	12.0	22.0	8.9	20.6
Insomnia	16.3	24.0	14.4	24.0	21.1	25.7	14.7	23.4	18.3	23.9	16.0	24.4	13.5	22.3
Appetite loss	3.4	12.1	2.2	8.4	4.9	16.5	3.0	11.7	2.7	10.3	3.7	10.5	5.0	16.0
Constipation	2.9	11.1	3.3	10.0	2.5	13.3	1.3	6.6	2.0	9.3	4.3	13.1	4.6	14.2
Diarrhoea	5.5	15.7	6.7	18.1	8.8	21.2	5.0	13.7	3.3	11.1	5.0	12.9	4.0	14.4
Financial difficulties	4.3	16.8	4.4	18.8	7.8	25.2	3.0	12.6	4.0	13.6	4.0	15.9	2.6	9.1
**Summary score**	90.7	11.0	90.9	10.3	89.1	14.6	91.5	11.1	90.5	9.6	90.8	11.1	91.5	9.3

^a^Mean scale scores were weighted by the sex and age distribution of the adult Dutch population, using the 2015 population distribution statistics published by the United Nations Department of Economic and Social Affairs Population Division

**Table 5:** Weighted EORTC QLQ-C30 scores^a^ for the Dutch general population stratified by age category (women)

	Full sample		18–29 years		30–39 years		40–49 years		50–59 years		60–69 years		70+ years	
	Mean	SD	Mean	SD	Mean	SD	Mean	SD	Mean	SD	Mean	SD	Mean	SD
**Global health/quality of life**	75.6	20.4	68.4	20.4	74.1	22.9	74.5	22.2	79.3	17.4	76.5	19.9	83.3	15.6
**Functioning scales**														
Physical	88.6	15.6	91.5	12.3	93.8	10.2	89.3	17.5	90.5	13.8	82.8	18.3	82.6	17.5
Role	87.6	22.6	89.0	23.4	88.1	21.9	86.5	25.6	89.4	20.0	83.8	24.5	88.2	19.2
Emotional	80.2	21.5	72.5	25.8	77.2	22.2	79.9	23.3	80.1	17.6	85.9	17.9	88.2	15.0
Cognitive	89.8	16.8	86.7	22.7	92.0	14.2	87.7	18.3	90.9	14.6	91.3	12.7	91.7	12.4
Social	90.7	19.2	89.0	19.9	90.5	20.3	87.3	24.2	94.1	13.9	91.3	17.4	92.3	17.7
**Symptom scales**														
Fatigue	26.8	24.8	35.6	28.6	27.2	22.3	29.4	26.8	22.8	20.2	24.2	24.7	18.4	19.4
Nausea/vomiting	4.8	14.1	9.1	20.3	6.0	14.7	3.7	9.7	2.5	9.6	3.7	12.7	2.5	11.2
Pain	21.2	24.8	22.0	24.4	17.0	23.0	19.2	25.7	23.9	24.2	25.5	28.0	19.0	23.1
Dyspnoea	11.7	21.3	12.1	19.0	10.7	25.5	9.3	20.2	11.6	19.7	14.3	25.7	12.0	18.7
Insomnia	26.2	27.1	23.5	25.3	23.2	23.7	29.7	32.1	29.0	29.3	28.3	25.7	23.7	24.8
Appetite loss	6.4	17.4	8.3	19.1	6.0	14.3	6.7	19.0	4.6	15.7	6.0	17.4	6.3	17.6
Constipation	6.9	15.4	6.1	16.4	7.1	15.2	7.0	15.9	7.9	15.0	5.7	14.3	7.7	15.6
Diarrhoea	8.2	19.5	12.1	22.7	10.1	21.0	7.0	18.5	5.9	16.6	7.7	20.6	5.3	15.5
Financial difficulties	5.4	17.4	9.1	25.1	3.0	9.6	4.7	14.3	6.6	17.0	5.3	16.3	3.0	13.5
**Summary score**	86.5	12.8	84.6	13.6	87.3	12.7	86.1	15.1	87.4	11.7	86.2	12.1	88.3	10.3

^a^Mean scale scores were weighted by the sex and age distribution of the adult Dutch population, using the 2015 population distribution statistics published by the United Nations Department of Economic and Social Affairs Population Division

The age patterns described above were found for both men and women. For physical functioning, scores were worse with increasing age (men: age 18–29 years: 96.2; age 70+ years: 87.9, Cohen’s d = 0.68; women: age 18–29 years: 91.5; age 70+ years: 82.6, Cohen’s d = 0.60), for emotional functioning, scores were better with increasing age (men: age 18–29 years: 77.2; age 70+ years: 92.7, Cohen’s d = 0.68; women: age 18–29 years: 72.5; age 70+ years: 88.2, Cohen’s d = 0.72). Varying patterns were found for the association between age and symptom experience.

## Discussion

This study describes Dutch EORTC QLQ-C30 normative data stratified by age and sex. Mean functioning ranged between 82 and 92 points (with 100 points indicating perfect functioning), while symptom scores ranged between 4 and 24 points (with 0 indicating no symptom burden). Clinically meaningful trends were found for emotional functioning (where scores were better with increasing age), similar to other recent publications in this area [15–18]. Most symptom scores remained stable over age categories. However, fatigue scores were better with increasing age (clinically meaningful difference). Men generally reported better functioning, and lower symptom burden for insomnia, pain, fatigue, and dyspnoea, but except for insomnia, these differences were not clinically meaningful.

Dutch general population data for the EORTC QLQ-C30 have been published previously by van de Poll-Franse et al. based on a 2009 survey of 1700 individuals [19]. Similar to our findings and the results of the larger European parent study from which our sample stems [13], van de Poll-Franse et al. described clinically meaningful worse physical functioning scores with increasing age; differences in scores between age groups for other functioning scales were not clinically meaningful. Like our findings, men scored significantly better than women did on most functioning scales but differences were not clinically meaningful [19]. Subsequently, Mols et al. [20] carried out five consecutive annual assessments (2009–2013) of the EORTC QLQ-C30 in 1743 (2009), 2050 (2010), 2040 (2011), 2194 (2012), and 2333 (2013) individuals from the Dutch general population. They reported that, in general, HRQoL was worse among the older age groups; these findings were clinically meaningful for physical and role functioning. Similar to our findings, they reported non-clinically meaningful higher summary scale scores in men than in women and in the younger compared to the older age groups. However, in contrast to our findings, Mols et al. found clinically meaningful age differences for pain and dyspnoea (both worse scores with increasing age), as well as clinically meaningful worse scores in women compared to men for fatigue, and pain.

Contrasting to van de Poll-Franse et al. [19] and Mols et al. [20], we found clinically meaningful better emotional functioning with increasing age. Previous international literature yields mixed findings, with some studies observing this trend [13, 16, 17, 30] and others not [31, 32]. Actual differences in emotional functioning may have occurred over time, for example by changes in society over the past decade that especially affected younger people. Further, both previous Dutch studies [19, 20] found a clinically meaningful worse fatigue scores with increasing age, while we found that fatigue scores were better in older respondents. Both previous Dutch studies reported clinically meaningful differences for other symptom scales, including pain, insomnia, and dyspnoea, while in our study we observed stable symptom scores. A possible explanation for the observed differences between studies could lie in the detrimental effect of health conditions on HRQoL [33]. In our study, 52% percent of individuals reported no chronic health conditions (weighted sample). This is higher than the 38% reported by van de Poll-Franse et al. [19], the 40% reported by Mols et al. [20], and the 41% of the actual proportion of individuals without chronic health conditions reported in a 2020 Dutch public health report [34]. In our study, in particular, participants in the older age categories may have been ‘healthier’ than the data from the Dutch public health report suggest, resulting in an overestimation of their HRQoL. A related Dutch report on national health statistics described relatively large differences in self-reported health experience between lower and more highly educated persons, with the latter reporting better health [35]. We included 42% of participants with a university degree or higher, whereas van de Poll-Franse et al. reported 37% and Mols et al. only 13%. According to Statistics Netherlands, that publishes annual data on the characteristics of the Dutch population, the percentage of individuals with a university degree was 16% in 2019 and has been increasing over the past decade across age groups [36].

Comparing our data to Statistics Netherlands 2017 suggests that the study data are representative in terms of most basic individual characteristics (age, sex, and marital status) [37]. Since 97.1% of the Dutch population had access to internet from their homes at the time we conducted our survey [38], it is unlikely that computer access played a role in the somewhat skewed educational background of our sample. A more plausible explanation is that highly educated individuals are more likely to participate in surveys than low-educated individuals, and it is therefore worthwhile to correct for education levels [39]; especially to avoid bias in the older age groups, as those who can use computers might be healthier because of their intellectual ability or access to health information [40]. Use of paper-and-pencil questionnaires and telephone-assisted surveys could aid in recruiting older individuals who would otherwise not be recruited into such studies. As opposed to age and sex, we could not correct for health status or education in our weighting approach, as that information was not available at an international standardised level, for instance from the United Nations statistics. In the original study by Liegl et al. [14], representativeness of sample characteristics was tested. Although the total sample was not representative based on educational level, as is the case with the present Dutch sample, it was found that observed group differences based on educational status were negligible, evidenced by very small effect sizes.

The mean functioning scores we report (between 82 and 92) are, together with scores from Austria (between 78 and 92 [16]), among the most favourable in Europe [13], where scores in Germany (between 74 and 85 [15]), Italy (between 74 and 88 [18]), and Spain (between 77 and 88 [17]) are slightly worse. In the international study sample and country-specific samples for Austria and Italy, HRQoL was generally better in respondents without health conditions [14, 16, 18]. Half of our population sample was free from health conditions (52%, weighted sample), comparable to the percentage reported in Austria (53%) [16]. In Germany (41%) [15], Italy (38%) [18], and Spain (39%) [17], and the parent sample (39%), notably smaller proportions of the population samples reported having no health conditions (weighted samples). Similar to our study, Lehmann et al. [16] reported that 35% of their sample completed university-level education while, in fact, only 15% of the Austrian population has that level of education. The core publication by Liegl et al. [14] investigated in depth the relationship between education and HRQoL. Post-compulsory compared to less than post-compulsory education was associated significantly with better HRQoL. However, differences were of low clinical relevance as indicated by the small effect sizes (all eta^2^ ≤ 0.015).

### Clinical practice implications and future research

In the current study, we observed floor effects for several symptoms, suggesting that a large proportion of the general population are not affected by these symptoms; hence, specific items of a cancer-specific questionnaire may not be applicable to them. However, the main aim of the current study was to provide a reference for users of the QLQ-C30 to enable them to put their cancer patients’ scores into context. Therefore, knowing that the prevalence of certain symptoms is very low in the general population is an important finding, too. Further, we are presenting mean scores in keeping with the other norm data publications [13–18]; however, an alternative presentation of the data may have been appropriate, too, for example, data presented as percentiles to show that there is a proportion of persons of the general population who are indeed experiencing symptoms and/or have lower functioning.

In clinical practice, both reference data from other cancer patients and general population normative data can aid in the interpretation of HRQoL data obtained from cancer patients. The importance of stratified comparison is underlined by the differences in functioning and symptoms we found between men and women, and between different age categories. Comparison with normative data is particularly interesting for determining the effect of cancer and cancer treatment on long-term survivorship: 60% of Dutch cancer patients will live at least five years after a cancer diagnosis [41]. The updated general population normative data presented here will enable such comparisons between the Dutch general population and Dutch cancer patients.

## Data Availability

The datasets analysed in this study are available upon request from the EORTC. Please use the Data Sharing form available through the EORTC website (https://www.eortc.org/data-sharing/).
